# WRKY Transcription Factors in *Nicotiana tabacum* Modulate Plant Immunity against Whitefly via Interacting with MAPK Cascade Pathways

**DOI:** 10.3390/insects12010016

**Published:** 2020-12-29

**Authors:** Dan-Mei Yao, Chi Zou, Yan-Ni Shu, Shu-Sheng Liu

**Affiliations:** Ministry of Agriculture Key Lab of Molecular Biology of Crop Pathogens and Insects, Institute of Insect Sciences, Zhejiang University, Hangzhou 310058, Zhejiang, China; wanttofly.cool@163.com (D.-M.Y.); zouchichi@126.com (C.Z.); shuyanni1991@163.com (Y.-N.S.)

**Keywords:** WRKY transcription factor, gene expression, plant defense, *Bemisia tabaci*, MAPK

## Abstract

**Simple Summary:**

WRKY transcription factors, a group of key regulators of many metabolic processes in plants, are now known to have a significant role in plant defense against sap-sucking insects such as aphids and whiteflies, but the molecular mechanisms underlying this plant defense is still poorly understood. In this study, we first showed that whitefly feeding and salicylic acid treatment of plants upregulate the expression of several WRKY transcription factors. We then showed that WRKYs’ contribution to plant defense is likely due to their interactions with the mitogen-activated protein kinase cascade (MAPK cascade) pathways in plants. Our findings contribute to a better understanding of the functions of WRKYs in plant defense against insect infestation.

**Abstract:**

WRKY transcription factors are key regulators of many plant processes, most notably coping with biotic and abiotic stresses. Recently, the function of WRKY in plant defense against phloem-feeding insects such as whitefly (*Bemisia tabaci*) has been brought to attention. In this study, we found that the expression levels of *Nicotiana tabacum WRKY4*, *WRKY6* and *WRKY10* were significantly upregulated when tobacco plants were infested with whiteflies or treated with salicylic acid. Compared to controls, whiteflies lived longer and laid more eggs on *NtWRKY*-silenced tobacco plants but performed less well on *NtWRKY*-overexpressing plants. The three NtWRKYs interacted with five mitogen-activated protein kinases (NtMAPKs) in vivo and in vitro. These results suggest that the WRKYs in tobacco positively modulate plant defense against whiteflies through interaction with the mitogen-activated protein kinase cascade (MAPK cascade) pathways, and thus provide new insights into plant defense against phloem-feeding insects.

## 1. Introduction

WRKY transcription factors are a large family of regulatory proteins ubiquitous in higher plants [1,2,3]. Members of the WRKY family contain at least one conserved WRKY domain consisting of a WRKYGQK motif at the N-terminal and a zinc-finger motif at the C-terminal. Based on the number of WRKYGQK motifs and structure of the zinc-finger motif, the WRKY superfamily can be classified into three groups, namely Group I, Group II, and Group III. Group I has two WRKY domains, while Group II and Group III each have only one WRKY domain. Group III WRKY has a unique zinc-finger motif, C2HC, which is distinct from C2H2 in the other two groups. Through interaction with other proteins such as mitogen-activated protein kinase (MAPK), mitogen-activated protein kinase kinase (MAPKK), and 14-3-3, WRKYs extensively modulate plant resistance against biotic stresses [4,5]. Salicylic acid (SA) and jasmonate acid (JA) are conserved positive regulators of plant defense [6]. In attempts to investigate the functions of WRKYs in modulating plant responses to insect attack, tobacco WRKY3 and WRKY6 were shown to coordinate responses to hornworm infestation and create induced resistance through regulation of the biosynthesis of phytohormones including jasmonates [7], and *WRKY 70* acted as an activator of SA-induced pathogenesis-related genes and a repressor of JA-responsive genes [8]. Furthermore, WRKY70 and WRKY72 were observed to contribute to the R gene *Mi-1*-mediated basal immunity against aphids in tomato [9,10].

The whitefly *Bemisia tabaci* is a species complex comprising likely >40 cryptic species, which include some important crop insect pests worldwide [11,12,13,14]. A few attempts have been made to investigate the role of WRKY in plant immunity against whiteflies. For example, Jin et al. (2016) showed that the expression levels of six *WRKY* genes in cotton plants were altered when plants were infested by whitefly; silencing *GhMPK3* led to enhanced susceptibility to whitefly on cotton plants via suppression of the MPK–WRKY–JA and ethylene (ET) pathways [15]. Wang et al. (2019) observed that Arabidopsis *AtWRKY33* negatively modulated plant defense against whitefly [16]. Huang et al. (2016) and Zhao et al. (2019) reported that the expression levels of tobacco *WRKY*s were altered following infection by whitefly-transmitted begomoviruses, and in turn plant resistance against whiteflies was suppressed [17,18]. These case studies indicate that WRKYs are widely involved in whitefly–plant interactions, and that their functions may vary with plants and many of the underlying molecular mechanisms are yet to be explored. 

The objective of this study was to further explore the roles of *Nicotiana tabacum WRKY*s (*NtWRKY*s) in plant immunity against whitefly. We first characterized the expression of *WRKY* genes upon whitefly infestation and phytohormone treatment, and performed bioassays of whiteflies on *NtWRKY*-silenced and *NtWRKY*-overexpressing tobacco plants. Using protein-binding assays in vivo and in vitro, we then examined the interactions between NtWRKYs and five tobacco MAPKs, wound-induced protein kinase (WIPK), salicylic acid-induced protein kinase (SIPK), NTF4-1, NTF4-2, and NRK1. The role of these MAPKs in plant defense against whitefly were also be confirmed. Our results reveal the importance of the interactions between WRKYs and MAPK cascade pathways in the modulation of plant immunity against whitefly and likely other phloem-feeding insects. 

## 2. Materials and Methods 

### 2.1. Plant and Whitefly

Tobacco plants, including *N. tabacum* cv. NC89, *N. benthamiana* line 16c, and *N. benthamiana* line H2B-RFP, were cultivated in climate chambers at 27 ± 2 °C under a 14 h light/10 h dark cycle with 50–70% humidity. The seeds of these plants were provided by the Institute of Biotechnology, Zhejiang University.

The Middle East-Asia Minor 1 species of the *Bemisia tabaci* whitefly complex was tested. A whitefly colony (*mtCOI* NCBI accession code: GQ332577) was originally established from a field whitefly sample collected in Hangzhou, China in 2009. The whitefly colony was maintained on tobacco plants (*N. tabacum* cv. NC89) in cages in a climate chamber at 27 ± 2 °C under a 14 h light/10 h dark cycle with 50–70% humidity. The purity of the whitefly colony was monitored by using the random amplified polymorphic DNA polymerase chain reaction (PCR) and sequencing the *mtCOI* gene every three to five generations [19].

### 2.2. Plasmid Construction and Transformation 

Coding sequences of *NtWRKY*s and *MAPK*s were amplified and cloned into pClone007 for sequencing (TSINGKE, Beijing, China). New constructs were generated using restriction endonuclease and T4 ligase. The primers of genes used are listed in Table S1. 

The new constructs used for expression in plants were transformed into *Agrobacterium tumefaciens* (EHA105) by electroporation. Recombinant plasmids used for prokaryotic protein expression were transformed into *Escherichia coli* (BL21) using a heat-shock method.

### 2.3. Virus-Induced Gene Silencing (VIGS) 

Virus-induced gene silencing (VIGS) was performed as previously described [20]. Fragments (about 300–600 bp) from target genes were amplified and cloned into a pBIN2mDNA1 vector. The new constructs were transformed into *A. tumefaciens* (EHA105). Agrobacterium cultures containing pBIN2mDNA1-NtWRKYs/MARKs and TBCSV infection clones were then co-infiltrated into *N. tabacum* plants at two-true-leaf stage. After 25 days, the transcription levels of *NtWRKY*s and *MAPK*s were determined by qRT-PCR.

### 2.4. Construction of Transgenic Plant

Full lengths of *NtWRKY*s were amplified and cloned into the pCHF3. The new constructs were transformed into *A. tumefaciens* (EHA105). The transgenic plants were generated by the Genvo Bio Company (Tianjin). The transcription levels of *NtWRKY*s were determined using qRT-PCR. 

### 2.5. Salicylic Acid (SA) and Jasmonate (JA) Treatments

Methyl jasmonate (MeJA) was dissolved in anhydrous ethanol at a concentration of 0.5 M, and SA was similarly dissolved in anhydrous ethanol at a concentration of 0.1 M. Plants were sprayed with 0.5 mM MeJA, 0.1 mM SA, or a mock solution containing 0.1% *v*/*v* ethanol such that the leaves were covered in a fine mist.

### 2.6. qRT-PCR and the Analysis of Relative Gene Expression Level

Total RNAs of plant leaves were isolated using the TRIzol method. cDNAs were synthesized using the PrimerScript RTTM reagent kit with gDNA eraser (Takara, Beijing, China). qPCRs were performed with SYBR Premix Ex TaqTMⅡ (Takara, China) using the BIO-RAD CFX96 PCR System (Bio-Rad, Hercules, CA, USA). *Glceraldehyde-3-phosphate dehydrogenase* (*GAPDH*) was selected as the reference gene. The relative expression levels of these genes were calculated using the 2^−ΔΔT^ method.

### 2.7. Observation of Whitefly Performance on Tobacco

In each replicate, 10 newly emerged whitefly adults (five males and five females) were released onto the abaxial surface of a leaf (enclosed by a clip cage) of a tobacco plant to feed, mate, and oviposit. Ten replicates (plants) were conducted for each treatment or control. Seven days after the release of the adults, the number of live female adults and the number of eggs on each leaf were counted. 

### 2.8. Bimolecular Fluorescent Complimentary (BiFC) Assay

Full lengths of *NtWRKY*s were amplified and cloned into the p2YN vector, and full lengths of *MAPKs* were amplified and cloned into the p2YC vector. The new constructs were transformed into *A. tumefaciens* (EHA105). Agrobacterium culture containing p2YN–NtWRKYs and p2YC–NtMAPKs were co-infiltrated into *N. benthamiana* line H2B-RFP. At 36–72 h postinoculation, fluorescence was examined by confocal microscope (Zeiss LSM710, Oberkochen, German).

### 2.9. Prokaryotic Recombinant Protein Expression

Full lengths of *NtWRKY*s were cloned into pMal-c5x vector, and full lengths of *NtMAPK*s were cloned into pGEX-6p-1. The new constructs were transformed into *E. coli* (BL21). Protein expression was induced for 24 h at 4 °C by adding 0.5 mM isopropyl-β-thiogalactopyranoside (IPTG). Cells containing MBP-NtWRKYs were collected and were purified using amylose resin (NEB), eluted with elution buffer (20 mM Tris-HCl, 200 mM NaCl, 1 mM DTT, 1 mM EDTA, 10 mM maltose, pH = 7.4), and desalted using disposable PD-10 desalting columns (GE Healthcare, Chicago, IL, USA). Cells containing GST-NtMAPKs were collected and purified using a Glutathione Sepharose 4 Fast Flow (GE Healthcare).

### 2.10. In Vitro Pull-Down Assay

A Glutathione Sepharose 4 Fast Flow containing GST-NtMAPKs was used to pull down MBP-NtWRKYs in vitro for 2 h at 4 °C. The beads were boiled with SDS loading buffer for 10 min. Proteins were separated using SDS-PAGE and detected using Western blot with an anti-MBP tag mouse monoclonal antibody (Abcam, Cambridge, UK). 

### 2.11. MAPK Activation Assay

Tobacco plants were infested by whiteflies at indicated time points. The leaves were then ground in the protein-extraction buffer according to previously described protocols [21]. Finally, the phosphorylated MAPKs were detected using Western blot with an anti-pTEpY antibody (Cell Signaling, Danvers, MA, USA). 

### 2.12. Statistical Analysis

Statistical significance of whitefly performance was determined using one-way ANOVA at a 0.05 level, followed by Fisher’s least-significant difference (LSD) tests. Percentage data of female survival were transformed by arcsine square root for statistical analysis, but the original data are presented for convenience of reading. qPCR data were analyzed using student’s *t* test at a significance level of *p* < 0.05. All analyses were performed using the software SPSS20.0 Statistics.

## 3. Results

### 3.1. Expression of NtWRKY4, NtWRKY6, and NtWRKY10 after Whitefly Infestation

**Figure 1:**
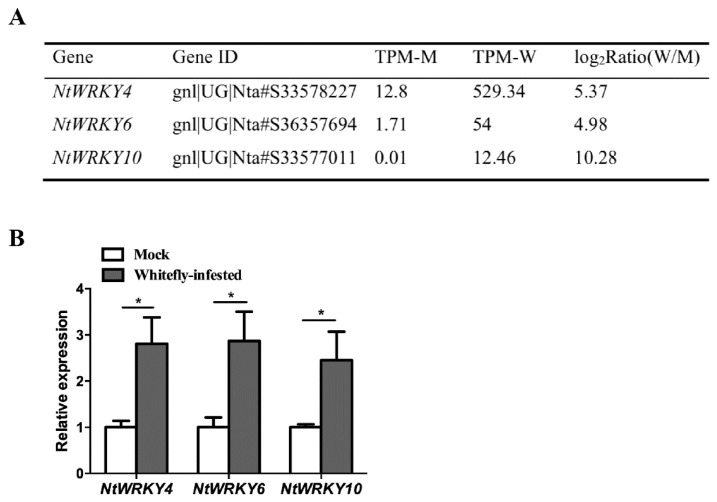
Whitefly infestation upregulated the expression levels of *NtWRKYs*. (**A**) Effect of whitefly feeding on expression of *NtWRKY4*, *NtWRKY6*, and *NtWRKY10* from DGE database. “M” denotes the tobacco plants mock-inoculated with agrobacterium, “W” denotes whitefly infestation. (**B**) qPCR validation of expression levels of *NtWRKYs* after whitefly infestation. The mock and whitefly-infested treatments in each of the three combinations were analyzed using Student’s *t* test at a significance of * *p* < 0.05.

Based on a published whitefly-infested tobacco digital gene expression (DGE) library (the accession no. GSE29812.), we found that the expression levels of *NtWRKY4*, *NtWRKY6*, and *NtWRKY10* were upregulated after whitefly infestation for 72 h (Figure 1A). We confirmed these results from DGE using qPCR analysis (Figure 1B). 

### 3.2. Expression of NtWRKY4, NtWRKY6, and NtWRKY10 after SA Treatment 

**Figure 2:**
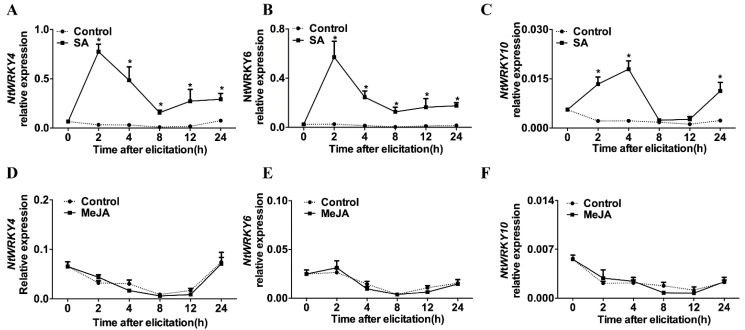
Expression levels of *NtWRKY4*, *NtWRKY6*, and *NtWRKY10* after salicylic acid (SA) and Methyl jasmonate acid (MeJA) treatments. (**A**–**C**) Effect of SA application on the expression of *NtWRKY4*, *NtWRKY6*, and *NtWRKY10*; (**D**–**F**) effect of MeJA application on the expression of *NtWRKY4*, *NtWRKY6*, and *NtWRKY10*. Three or four replicates were conducted per treatment. The expression levels of the control and SA (or MeJA) treatment at each time point were analyzed using Student’s *t* test at a significance of * *p* < 0.05.

The SA treatment, but not MeJA treatment, induced the expression of *NtWRKY4*, *NtWRKY6*, and *NtWRKY10* (Figure 2). *NtWRKY4* and *NtWRKY6* reached their expression peak at 2 h after SA treatment, while *NtWRKY10* reached its expression peak at 4 h after SA treatment, implying that *NtWRKY4*, *NtWRKY6*, and *NtWRKY10* may be regulated by the SA signaling pathway.

### 3.3. NtWRKY4, NtWRKY6, and NtWRKY10 Positively Regulated Plant Defense against Whiteflies

**Figure 3:**
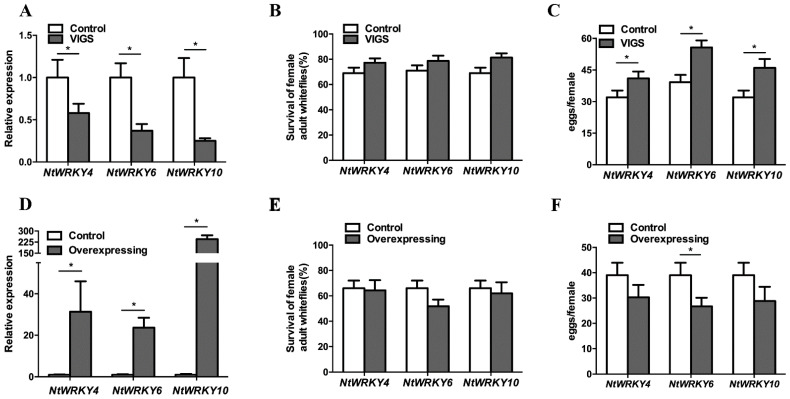
*NtWRKY4*, *NtWRKY6*, and *NtWRKY10* positively regulated tobacco defense against whiteflies. (**A**) Expression levels of *NtWRKY4*, *NtWRKY6*, and *NtWRKY10* in silenced plants. (**B**) Survival rates of whiteflies on *NtWRKY4*-, *NtWRKY6*-, and *NtWRKY10*-silenced plants. (**C**) The fecundities of whiteflies on *NtWRKY4*-, *NtWRKY6*-, and *NtWRKY10*-silenced plants. (**D**) Expression levels of *NtWRKY4*, *NtWRKY6*, and *NtWRKY10* in transgenic overexpressed plants. (**E**) Survival rates of whiteflies on *NtWRKY4*-, *NtWRKY6*-, and *NtWRKY10*-overexpressing plants and controls. (**F**) The fecundities of whiteflies on *NtWRKY4*-, *NtWRKY6*-, and *NtWRKY10*-overexpressing plants. The gene expression levels of the control and VIGS (or overexpressing) plants in each of the treatments was analyzed using Student’s *t* test at a significance of * *p* < 0.05; the percentages of whitefly survival among the three controls and VIGS (or overexpressing) plants were analyzed using one-way ANOVA at a significance of * *p* < 0.05; similarly the numbers of eggs laid per female among the three controls and VIGS (or overexpressing) were analyzed using one-way ANOVA at a significance of * *p* < 0.05.

To investigate the function of the three NtWRKYs in tobacco defense against whiteflies, we silenced and overexpressed these genes via VIGS and transgenic overexpression techniques, respectively. qPCR analysis indicated that the VIGS method downregulated the expression levels of the three *NtWRKYs* (Figure 3A). While silencing the expression levels of the three *NtWRKYs* did not affect the survival of female whiteflies, the number of eggs laid by the females on *NtWRKY*-silenced plants increased significantly compared to control plants (Figure 3B,C). Meanwhile, transgenic overexpression increased the expression levels of *NtWRKY4*, *NtWRKY6*, and *NtWRKY10* 14.7-, 4.8-, and 26.2-fold, respectively (Figure 3D). While this overexpression did not affect the survival, the numbers of eggs laid by the females was reduced significantly on transgenic *NtWRKY6*-overexpressed plants compared to control plants (Figure 3E,F). These results indicate that NtWRKY4, NtWRKY6, and NtWRKY10 positively regulate the plant defense against whiteflies.

### 3.4. MAPK Interacted with NtWRKY4, NtWRKY6, and NtWRKY10

**Figure 4:**
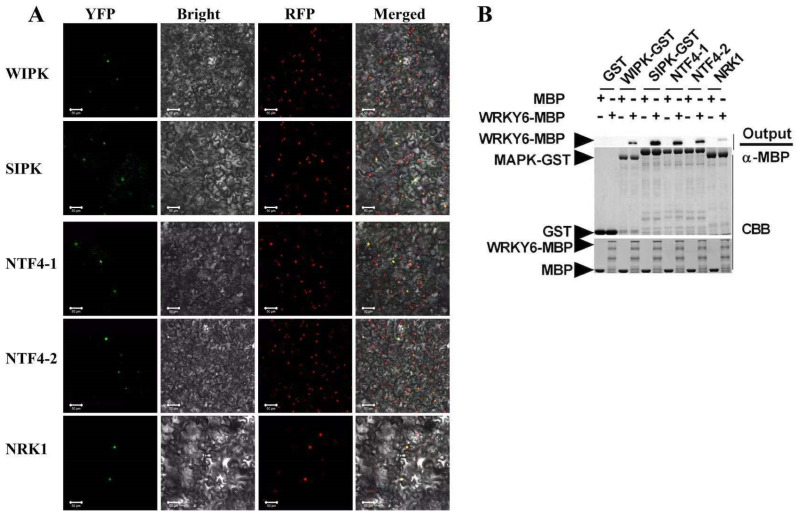
The interactions between NtWRKY6 and five tobacco MAPKs, wound-induced protein kinase (WIPK), salicylic acid-induced protein kinase (SIPK), NTF4-1, NTF4-2, and NRK1. (**A**) In vivo interaction between WRKY6 and NtMAPKs as shown by BiFC analysis. NtWRKY6-YFPN and NtMAPKs-YFPC were transiently co-expressed in *N. benthamiana* line H2B-RFP, of which the nuclei were marked with RFP fusion protein. Photos were imaged at 48 h using a Zeiss LSM710 confocal microscope. Columns from left to right represent YFP fluorescence, bright field, RFP fluorescence, and YFP/RFP/bright field overlay. Scale bars: 50 μm. (**B**) In vitro interaction between NtWRKY6 and NtMAPKs as shown by pull-down assay. Proteins GST, NtMAPKs-GST, MBP, and NtWRKY6-MBP were expressed via prokaryotic expression, and purified using glutathione agarose beads or Amylose resin. GST or NtMAPK-GST fusion proteins were used to pull down MBP or NtWRKY6-MBP fusion proteins. Binding proteins were analyzed via SDS-PAGE and Western blot assays using anti-MBP antibodies. At the start, samples (Input) were stained with Coomassie blue solution. GST and MBP proteins were used as negative controls.

MAPK is known to locate upstream of the WRKY transcription factor and regulate the expression of the WRKY at the transcriptional level and post-transcriptional level [22,23,24,25]. In tobacco, WIPK, SIPK, NTF4-1, NTF4-2, and NRK1 in MAPK pathway have been reported to regulate plant defense response [26,27]. The results of the BiFC assay here indicated that NtWRKY4 interacted with WIPK, SIPK, NTF4-1, and NTF4-2 (Supplementary Figure S1A), while NtWRKY6 and NtWRKY10 interacted with WIPK, SIPK, NTF4-1, NTF4-2, and NRK1 (Figure 4A; Supplementary Figure S1B). GST pull-down assay also showed that NtWRKY4, NtWRKY6, and NtWRKY10 interacted with five MAPK proteins (Figure 4B; Supplementary Figure S1C,D). Overall, the data suggest that whitefly infestation likely activated the plant MAPK pathway, which regulates WRKY transcription factors to enhance plant resistance against whitefly.

### 3.5. Silencing MAPKs Improved Whitefly Performance on Tobacco Plants

**Figure 5:**
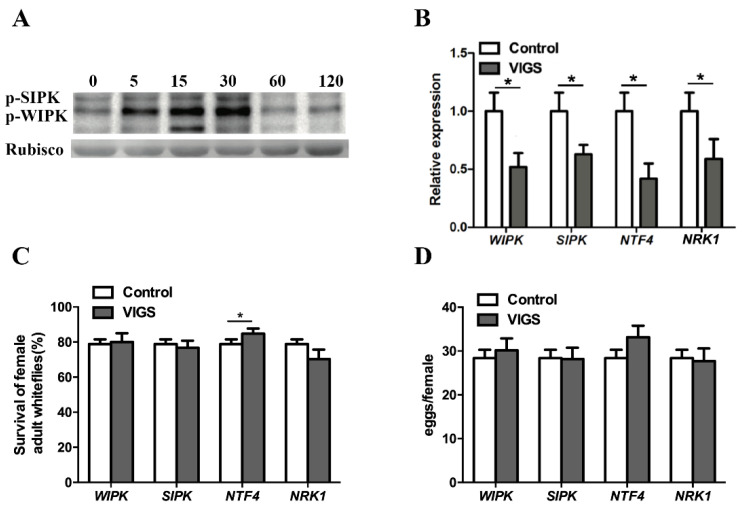
Effect of *wound-induced protein kinase* (*WIPK*), *salicylic acid-induced protein kinase* (*SIPK*), *NTF4*, and *NRK1* VIGS on the performance of whiteflies. (**A**) Whitefly feeding activated SIPK and WIPK. About 1000 whiteflies were released onto each plant, and samples were collected at 0, 5, 15, 30, 60, 120 min. Activated SIPK and WIPK were tested using Western blot with an anti-pTEpY antibody. (**B**) The expression levels of *WIPK*, *SIPK*, *NTF4*, and *NRK1* in gene-silenced tobacco plants and controls. (**C**) Survival rate of whiteflies on *WIPK*-, *SIPK*-, *NTF4*-, and *NRK1*-VIGS plants and controls. (**D**) The fecundity of whiteflies on *WIPK*-, *SIPK*-, *NTF4*-, and *NRK1*-VIGS plants and controls. As *NTF4-1* and *NTF4-2* have 98% sequence similarities, we used a common region for silencing and qPCR of these two genes. *NTF4* represents *NTF4-1* and *NTF4-2*. The two gene expression levels of control and VIGS in each of the four combinations in (**B**) were analyzed using Student’s *t* test at a significance of * *p* < 0.05; the percentages of whitefly survival (**C**) and the mean numbers of eggs laid per female (**D**) among the four control and VIGSs were analyzed using one-way ANOVA at a significance of * *p* < 0.05.

MAPK always function in the upstream of the WRKY family [8,28,29]. As NtWRKY4, NtWRKY6, and NtWRKY10 all positively regulate tobacco plants against whiteflies and can interact with the five MAPKs, we were curious about the function of the five MAPKs in tobacco plants. Our data indicated that whitefly infestation activated the MAPK pathway, especially at 15–30 min following the initiation of whitefly infestation (Figure 5A). We downregulated the expression levels of the five *MAPK*s using VIGS (Figure 5B). The results indicated that silencing *NTF4-1* and *NTF4-2* together marginally improved the survival of females, but did not affect the number of eggs laid by the females; silencing the remaining three *MAPK*s did not affect survival or number of eggs laid by the females (Figure 5C,D). 

## 4. Discussion

In this study, we first confirmed the induction of expression of *NtWRKY4*, *NtWRKY6*, and *NtWRKY10* by whiteflies. Silencing or overexpressing *NtWRKY*s in tobacco plants manipulated the performance of whiteflies. Wang et al. (2019) reported that WRKY33 from Arabidopsis is an important component of plant anti-whitefly resistance, and that it interacts with Bsp9, an effector from whiteflies [14]. Our data seemed also to suggest that whitefly effectors may directly or indirectly interact with WRKYs from crops like tobacco to modulate plant immunity.

The MAPK pathway of plants can be activated when plants are attacked by pathogens or insects [21,30]. Activated MAPK can phosphorylate WRKY and trigger downstream signaling pathways [8,28,29]. The data from this study also suggest that whitefly infestation phosphorylates SIPK and WIPK. Both BiFC and GST pull-down assays confirmed the interaction between MAPKs and WRKYs. Cosilencing *NTF4-1* and *NTF4-2* enhanced the performance of the whitefly on tobacco plants. These five MAPKs have high similarities in protein sequences, which may explain why whitefly fitness did not change on single-*MAPK*-silenced plants. We thus infer that whitefly infestation could activate the MAPK pathway, which phosphorylates WRKYs to induce the defense reaction of the tobacco plants. However, which one or ones of MAPKs can phosphorylate WRKY warrants further investigation.

Many studies have shown that the function of the WRKY family is often related to plant defense-associated phytohormones such as the SA and JA signaling pathways [8,31,32]. Previous work has shown that whitefly infestation can induce SA response, and SA can induce the expression of *WRKY*s [33]. Our findings seem to provide another case study in which NtWRKYs and SA signaling are connected. Future effort may be attempted to reveal the detailed mechanisms underlying the interactions between NtWRKYs and SA signaling to better understand the roles of WRKY in mediating plant defense against biotic stresses, including insect infestation.

## 5. Conclusions

Plants have evolved various ways to become less hospitable to insect herbivores. WRKY transcription factors are key regulators of many plant processes, most notably coping with biotic and abiotic stresses. In this study, the expression levels of tobacco WRKY transcription factors (*NtWRKY4*, *NtWRKY6*, and *NtWRKY10*) were significantly upregulated when the plants were infested by whitefly and treated with salicylic acid. Silencing the expression levels of *NtWRKY*s in plants significantly increased the number of eggs laid by the whiteflies compared to the control plants. The numbers of eggs laid by the females was reduced significantly on transgenic *NtWRKY6*-overexpressing plants compared to the control plants. Furthermore, whitefly infestation activated the MAPK pathway, and the three NtWRKYs interacted with NtMAPKs (WIPK, SIPK, NTF4-1, NTF4-2, and NRK1) in vivo and in vitro. These results suggest that the WRKYs in tobacco positively modulate plant defense against whiteflies by interacting with MAPK cascade pathways. Our findings provide new evidence of the interaction between NtWRKYs and SA signaling, and help to improve our understanding of the roles of WRKY in mediating plant defense against biotic stresses, including insect infestation.

## Data Availability

Data sharing not applicable.

## Supplementary Materials

The following are available online at https://www.mdpi.com/2075-4450/12/1/16/s1: Table S1. Primers used in this study. Figure S1. The interaction between NtWRKYs and five tobacco MAPKs, wound-induced protein kinase (WIPK), salicylic acid-induced protein kinase (SIPK), NTF4-1, NTF4-2, and NRK1. (**A**,**B**) In vivo interaction between WRKY4, WRKY10 and NtMAPKs as shown by BiFC analysis. NtWRKYs-YFPN and NtMAPKs-YFPC were transiently co-expressed in *Nicotiana benthamiana* line H2B-RFP, of which nucleus were marked with RFP fusion protein. Photos were imaged at 48 h using a Zeiss LSM710 confocal microscope. Columns from left to right represent YFP fluorescence, bright field, RFP fluorescence, and YFP/RFP/bright field overlay. Scale bars 50μm. (**C**,**D**) In vitro interaction between NtWRKYs and NtMAPKs as shown by pull-down assay. Proteins GST, NtMAPKs-GST, MBP, NtWRKYs-MBP were expressed by prokaryotic expression, and purified by Glutathione agarose beads or Amylose resin. GST or NtMAPKs-GST fusion proteins were used to pulldown MBP or NtWRKYs-MBP fusion proteins. Binding proteins were analyzed via SDS-PAGE and western blot assays using anti-MBP antibodies. At the start of samples (Input) were stained by Coomassie blue solution. GST and MBP proteins were used as negative controls.
