# Supplementary material for: WRKY Transcription Factors in Nicotiana tabacum Modulate Plant Immunity against Whitefly via Interacting with MAPK Cascade Pathways
Source: Insects. 2020 Dec 29;12(1):16. doi: 10.3390/insects12010016 (PMC7824566; doi:10.3390/insects12010016)
Supplement: Supplementary file 1 [file insects-12-00016-s001.pdf]

Table S1. Primers used in this paper.

gene	Primer name	Primer sequence	Primer application
NtWRKY4	WRKY4-qPCR-F	CACTGAAAGATCGACGAGGA	qRT-PCR
	WRKY4-qPCR-R	TTGGCATTGAGAATGTGCTT	qRT-PCR
NtWRKY6	WRKY6-qPCR-F	CAAGAAACCTCTTGCAACCA	qRT-PCR
	WRKY6-qPCR-R	TTAACTTCGGCGGTATCCTC	qRT-PCR
NtWRKY10	WRKY10-qPCR-F	GTCCGTTGTTTCTGACCCTT	qRT-PCR
	WRKY10-qPCR-R	CCGTCCTGGTAACTTCTGCT	qRT-PCR
NtWIPK	WIPK-qPCR-F	AACTCACGGCGGACAATA	qRT-PCR
	WIPK-qPCR-R	CCATAAGCACCACGACCA	qRT-PCR
NtSIPK	SIPK-qPCR-F	CCGGTGGCCGGTATGGATAA	qRT-PCR
	SIPK-qPCR-R	CAAACGATGCCGTAAGCACCT	qRT-PCR
NtNTF4	NTF4-qPCR-F	CCGGTGGCCGGAATCGATAA	qRT-PCR
	NTF4-qPCR-R	CGATGGGCATAATCGGTGGCT	qRT-PCR
NtNRK1	NRK1-qPCR-F	ACATCCCTCCTATTCAACCTGTCG	qRT-PCR
	NRK1-qPCR-R	ACCTCCTCCTTCGTCTCCGA	qRT-PCR
NtGAPDH	GAPDH-qPCR-F	GCAGTGAACGACCCATTTATCTC	qRT-PCR
	GAPDH-qPCR-R	AACCTTCTTGGCACCACCCT	qRT-PCR
NtWRKY4	siWRKY4-F	CGCGGATCCAACCATTAACGAAACCTC	VIGS
	siWRKY4-R	TGCTCTAGACACCTATTCTGAACCACAT	VIGS
NtWRKY6	siWRKY6-F	CGCGGATCCTCCACTTATCCAAGGCACT	VIGS
	siWRKY6-R	TGCTCTAGACGGAGGCTTCAAATACTG	VIGS
NtWRKY10	siWRKY10-F	CGCGGATCCGCAGCGAGAAGAAGGAAG	VIGS
	siWRKY10-R	TGCTCTAGAGCCCACAGCGATAGGTTA	VIGS
NtWIPK	siWIPK-F	CGCGGATCCCTTCATAGAGATCTCAAACC	VIGS
	siWIPK-R	TGCTCTAGATTCTGTTGGGGTGCCAAGAA	VIGS
NtSIPK	siSIPK-F	CGCGGATCCTTGATTGGTACTCCTTCAGA	VIGS
	siSIPK-R	TGCTCTAGACATCTGTTCCTCCGTAAGGG	VIGS
NtNTF4	siNTF4-F	CGCGGATCCTCTGCTTATGGAGTTGATTG	VIGS
	siNTF4-R	TGCTCTAGAGTAAGGGCATGCTGTTCAAA	VIGS
NtNRK1	siNRK1-F	CGCGGATCCTTAATGGATACTGATCTGCA	VIGS
	siNRK1-R	TGCTCTAGAAATTGCTGCAGTATATTCAG	VIGS
NtWRKY4	OeWRKY4-F	CGAGCTCATGGAGCCTGAAAATTACAC	Transgenic overexpression
	OeWRKY4-R	GCTGCAGTTAAAATTCAAAAGATAAATC	Transgenic overexpression
NtWRKY6	OeWRKY6-F	CGAGCTCATGGAGTCTCCGTTGCCGGAA	Transgenic overexpression
	OeWRKY6-R	GCTGCAGTCAAGAATTGTACCCTTCAAA	Transgenic overexpression
NtWRKY10	OeWRKY10-F	GGGATCCATGTACACCATGCGAATTCAG	Transgenic overexpression
	OeWRKY10-R	GCTGCAGTCAGAACTGCGAAGGACCTTG	Transgenic overexpression
NtWRKY4	WRKY4-nYFP-F	CCCTTAATTAACATGGAGCCTGAAAATTACAC	BiFC
	WRKY4-nYFP-R	GGGACTAGTAAATTCAAAAGATAAATC	BiFC
NtWRKY6	WRKY6-nYFP-F	CCCTTAATTAACATGGAGTCTCCGTTGCCGGAA	BiFC
	WRKY6-nYFP-R	GGGACTAGTAGAATTGTACCCTTCAAA	BiFC
NtWRKY10	WRKY10-nYFP-F	CCCTTAATTAACATGTACACCATGCGAATTCAG	BiFC
	WRKY10-nYFP-R	GGGACTAGTGAACTGCGAAGGACCTTG	BiFC
NtWIPK	WIPK-cYFP-F	CCCTTAATTAACATGGCTGATGCAAATATGGG	BiFC
	WIPK-cYFP-R	GGGACTAGTAGCATATTCAGGATTCAGTGA	BiFC
NtSIPK	SIPK-cYFP-F	CCCTTAATTAACATGGATGGTTCTGGTCAGCA	BiFC
	SIPK-cYFP-R	GGGACTAGTCATATGCTGGTATTCAGGAT	BiFC

NtNTF4	NTF4-cYFP-F	CCCTTAATTAACATGGATGGTCCAGCTCATCA	BiFC
	NTF4-cYFP-R	GGGACTAGTCATGTGCTGGTATTCAGGAT	BiFC
NtNRK1	NRK1-cYFP-F	CCCTTAATTAACATGGAAAACGAAACCAATGA	BiFC
	NRK1-cYFP-R	GGGACTAGTCTTCATTGTATTAGGATCAA	BiFC
NtWRKY4	WRKY4-MBP-F	TTGCGGCCGCATGGAGCCTGAAAATTACAC	prokaryotic expression
	WRKY4-MBP-R	CGGGATCCTTAAAATTCAAAAGATAAATC	prokaryotic expression
NtWRKY6	WRKY6-MBP-F	TTGCGGCCGCATGGAGTCTCCGTTGCCGGAA	prokaryotic expression
	WRKY6-MBP-R	CGGGATCCTCAAGAATTGTACCCTTCAAA	prokaryotic expression
NtWRKY10	WRKY10-MBP-F	TTGCGGCCGCATGTACACCATGCGAATTCAG	prokaryotic expression
	WRKY10-MBP-R	CGGGATCCTCAGAACTGCGAAGGACCTTG	prokaryotic expression
NtWIPK	WIPK-GST-F	CGGGATCCATGGCTGATGCAAATATGGG	prokaryotic expression
	WIPK-GST-F	CCCCCGGGTTAAGCATATTCAGGATTCA	prokaryotic expression
NtSIPK	SIPK-GST-F	CGGGATCCATGGATGGTTCTGGTCAGCA	prokaryotic expression
	SIPK-GST-F	CCCCCGGGTCACATATGCTGGTATTCAG	prokaryotic expression
NtNTF4	NTF4-GST-F	CGGGATCCATGGATGGTCCAGCTCATCA	prokaryotic expression
	NTF4-GST-F	CCCCCGGGTCACATGTGCTGGTATTCAG	prokaryotic expression
NtNRK1	NRK-GST-F	CGGGATCCATGGAAAACGAAACCAATGA	prokaryotic expression
	NRK-GST-R	CCCCCGGGTCACTTCATTGTATTAGGAT	prokaryotic expression

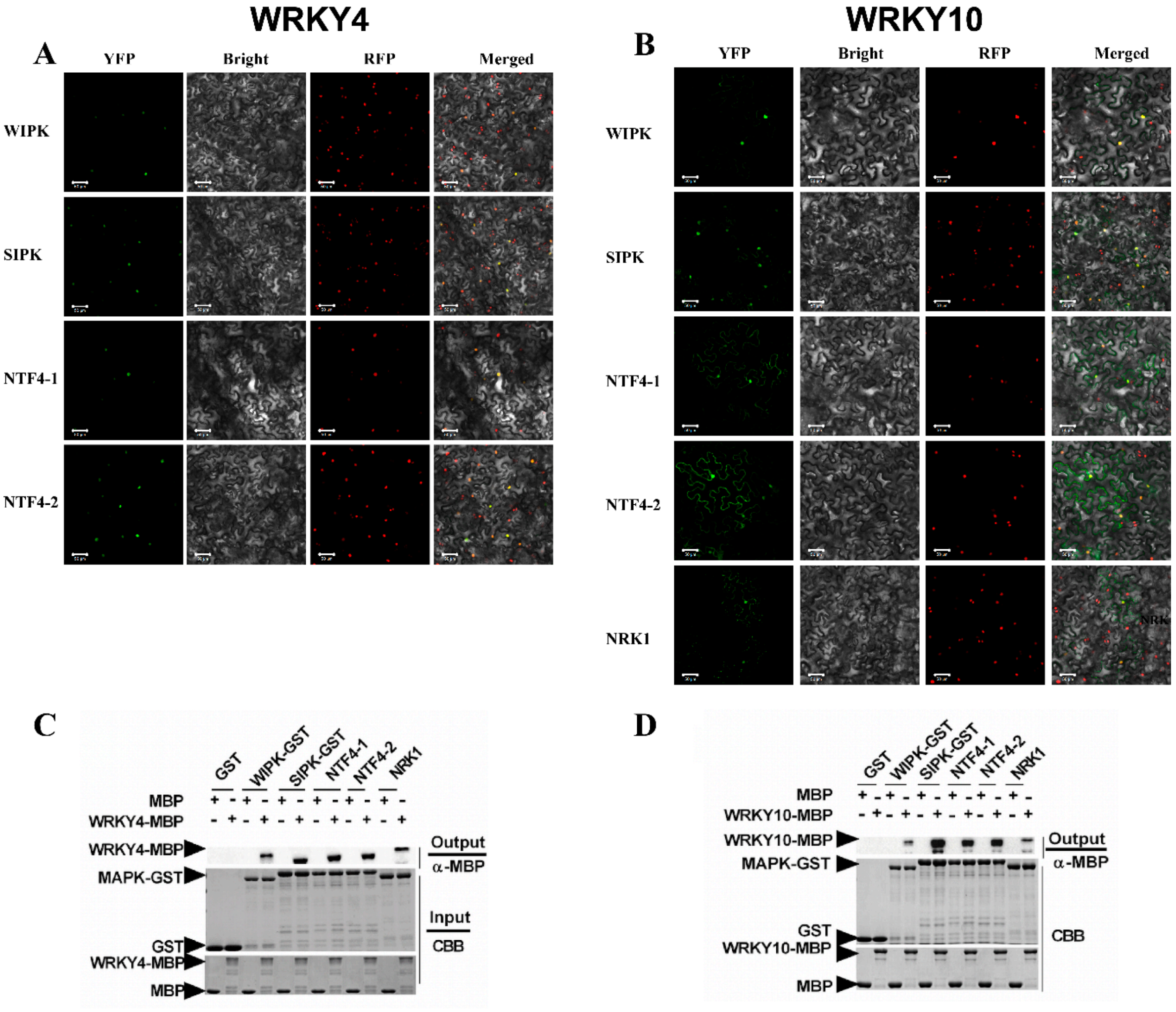

Figure S1. The interaction between NtWRKYs and five tobacco MAPKs, wound-induced protein kinase (WIPK), salicylic acid-induced protein kinase (SIPK), NTF4-1, NTF4-2, and NRK1. **(A, B)** *In vivo* interaction between WRKY4, WRKY10 and NtMAPKs as shown by BiFC analysis. NtWRKYs-YFPN and NtMAPKs-YFPC were transiently co-expressed in *Nicotiana benthamiana* line H2B-RFP, of which nucleus were marked with RFP fusion protein. Photos were imaged at 48 h using a Zeiss LSM710 confocal microscope. Columns from left to right represent YFP fluorescence, bright field, RFP fluorescence, and YFP/RFP/bright field overlay. Scale bars 50μm. (**C, D**) *In vitro* interaction between NtWRKYs and NtMAPKs as shown by pull-down assay. Proteins GST, NtMAPKs-GST, MBP, NtWRKYs-MBP were expressed by prokaryotic expression, and purified by Glutathione agarose beads or Amylose resin. GST or NtMAPKs-GST fusion proteins were used to pulldown MBP or NtWRKYs-MBP fusion proteins. Binding proteins were analyzed via SDS-PAGE and western blot assays using anti-MBP antibodies. At the start of samples (Input) were stained by Coomassie blue solution. GST and MBP proteins were used as negative controls.
